# Analysis of factors influencing innovative behaviors of intensive care unit nurses using a random forest model-a multicentre cross-sectional study

**DOI:** 10.1186/s12912-025-04052-2

**Published:** 2025-11-17

**Authors:** Wen-Jie Ge, Zhi-Min Cao, Xin-Yi Zhu, Shou-Jun Zhu

**Affiliations:** Department of Nursing & Department of Intensive Care Unit, The First Affiliated Hospital of USTC, Division of Life Sciences and Medicine, University of Science and Technology of China, 17 Lujiang Road, Luyang District, Hefei, Anhui Province 230001 China

**Keywords:** Spiritual climate, Information literacy, Thriving at work, Innovative behaviour, Intensive care, Nursing, Random forest model, Lasso

## Abstract

**Aim:**

To identify and rank the key determinants-including sociodemographic factors, Spiritual Climate, Information Literacy, and Thriving at Work-of Innovative behaviours among intensive care nurses, and provide a theoretical basis for targeted interventions.

**Background:**

Nurse innovation behavior is essential for the development of the nursing discipline; yet, significant progress in enhancing the innovation behavior of Intensive care nurses has been limited over the past few decades. Nurses must identify deficiencies and areas for improvement within the intensive medical care system while performing care practices. For nurses to identify solutions to these issues through an innovative approach, they must adopt innovative behaviors.

**Methods:**

A multicentre cross-sectional study was conducted. We gathered data from a convenient sample of 587 Intensive care nurses from twenty-three hospitals of grade 2 A and above hospital in Anhui, China. The questionnaire included questions on participant demographic information, the Thriving at Work Scale, the Information Literacy Scale, the Spiritual Climate Scale, and the Innovative Behavior Inventory. Lasso-logistic regression was employed to identify influencing factors, and a random forest model was used to rank the importance of variables.

**Results:**

The total score of innovative behavior of Intensive care nurses was 76.43 ± 14.67, and the results of LASSO analysis showed that the error was the smallest when the value of λ was 1.531. The number of influential factors corresponding to it was 6. The variables that ranked the top 6 in importance in the random forest model were included in the multifactorial logistic regression analysis. The results showed that spiritual climate, information literacy, thriving at work, the degree of love for nursing work, education level, and age were the main influences on the innovative behavior of Intensive care nurses (all *P* < 0.05), explaining a total of 55.7% of the variation, with spiritual climate being the primary influence.

**Conclusion:**

Intensive care nurses demonstrated moderately high levels of innovative behaviour. To further enhance innovation capacity, nursing managers should implement tiered information literacy training programs, improve the spiritual climate in Intensive Care Unit environments, facilitate nurse participation in multi-dimensional thriving at work interventions, and encourage highly educated and senior nurses to leverage their strengths.

## Introduction

Amid rapid advancements in health technology and rising healthcare demands, hospitals must enhance their efficiency, innovation capabilities, and value [1]. The sustainable development and innovation of healthcare organizations depend on building innovative healthcare teams [2]. As essential members of these teams, clinical nurses possess unique work characteristics—sustained patient contact and the delivery of comprehensive care—enabling them to identify opportunities for improvement in clinical practice and thus drive innovation to enhance healthcare service quality [3]. Nurses, who represent the largest segment of the global healthcare workforce, play a crucial role in advancing healthcare innovation [4]. Nurses’ innovative behavior refers to the process of nurses’ behavior based on the needs of health promotion and disease prevention and control, through exploring and developing innovative nursing technologies and practice models, introducing and applying them to their work under the mechanism of collaborative cooperation, which mainly consists of 3 stages: generating ideas, obtaining support, and realizing ideas [5, 6]. Asurakkody et al. [7] reported that the consequences of innovative behavior included increased job productivity, lower levels of job burnout, higher job satisfaction, improved ability to solve organizational problems, increased organizational commitment, and enhanced organizational efficiency and effectiveness. A recent study emphasised that the innovative behavior of nurses plays a significant role in shaping their productive performance [8]. The American Nursing Association’s “2024 ANA Enterprise Strategic Plan” lays out a goal to raise nursing’s professional standards globally, with an emphasis on developing nurses who can use creative nursing techniques in complex and dynamic settings to improve public health outcomes [9]. Meantime, China’s National Health Commission has issued the ‘National Nursing Development Plan (2021–2025)‘ [10], focusing on the new expectations of the people in the field of nursing, grasping the characteristics of nursing work, innovating the nursing service model, and striving to promote the reform and development of the nursing service industry. The intensive care unit (ICU) is a specialized department that provides care and treatment for the most critically ill patients due to its advanced and unique facilities. With the rapid advancements in science and technology, the ICU has evolved into a highly specialized field, creating a complex and often stressful work environment [11]. The inherent complexity of care in these units makes them susceptible to errors and adverse events. Epidemiological data indicate that ICUs report the highest incidence rates of adverse events among hospital departments, with prevalence rates ranging from approximately 17% to 31% for incidents such as pressure ulcers, patient falls, and hospital-acquired infections [12]. Research has demonstrated that innovation in intensive care nursing plays a crucial role in improving access to healthcare services, enhancing care quality, and increasing treatment efficiency and productivity [13]. Consequently, for ICU nurses, innovative behavior is not an optional ‘add-on’ but a core professional competency essential to safeguard patient safety and enhance the quality of care in high-stakes situations.

“Innovation in intensive care nursing” can be defined as the application of novel ideas, techniques, or procedures to address the critical needs of individuals, reduce healthcare costs, and enhance efficiency [14]. The innovative behavior of ICU nurses is positively associated with higher-quality care, as shown in various studies, mitigating complications, improving patient outcomes, and shortening hospitalization duration, thereby contributing to a more comfortable recovery experience [15–17]. However, despite its critical importance, the level of innovative behavior among ICU nurses remains insufficient. A national survey conducted in China by Gao et al. (2016) revealed that only 12.00% of ICU nurses demonstrated strong innovative behaviors, with the majority adhering to fixed nursing procedures and engaging in limited technical innovation [18]. This underscores a notable gap between the demand for innovation in critical care environments and nurses’ current levels of innovation. Compounding this issue, while innovative behavior in the general nursing population has been studied extensively, there remains limited research focused specifically on the unique cohort of intensive care nurses [19, 20].

Therefore, given the critical role of innovation in ICU settings and the identified gaps in both practice and research, it is both valuable and necessary to conduct a focused investigation into the factors influencing innovative behavior among intensive care nurses, with the ultimate aim of developing targeted strategies to enhance it. Several sociodemographic factors [6], such as gender, age, position, marital status, health status, and level of education, have been suggested to influence nurses’ innovative behavior; however, the results of studies of the effects of demographic characteristics on nurses’ innovative behavior have been mixed. Li et al. (2025) found no significant effect of age or position on nurses’ innovative behavior [21], but another study reported that these factors were associated with innovative behavior [22]. These findings indicate that the factors affecting innovative behavior warrant further investigation and offer guidance for identifying potential variables.

## Literature review

Antecedents to innovative behavior, comprising organizational, work environment, and individual characteristics, represent key factors affecting innovation [7]. Within these domains, nurses’ information literacy [23, 24], thriving at work [25], and spiritual climate [12, 26, 27] constitute critical variables, each representing a distinct level of influence on innovative behavior (individual, organizational, and work environment).

For individuals, information literacy is the foundation for lifelong learning and contributing to the knowledge society, encompassing the comprehensive performance of individuals in effectively acquiring and utilizing information in conjunction with their own needs [28]. Given the significance of innovative behavior among Intensive care nurses, information literacy—as an individual characteristic—is recognized as a key factor in generating innovative ideas to enhance such behavior. Information literacy refers to the ability of nursing professionals to accurately and comprehensively retrieve, objectively evaluate, and effectively apply information to resolve clinical dilemmas [29]. Information literacy serves as a critical mediating factor between workplace learning determinants and innovative outcomes, thereby enhancing both knowledge creation and the development of practical solutions [23]. Wang et al. (2025) demonstrated that Information Literacy positively influences the innovative behaviors of clinical nurses [24]. Fundamentally, enhanced information literacy among ICU nurses signifies their advanced capabilities in acquiring, managing, and effectively utilizing information. This proficiency serves as a catalyst for innovation and the development of valuable clinical resources, thereby fostering greater awareness of innovation and strengthening systemic innovation capacity within critical care settings. Advancing information literacy enables ICU nurses to translate evidence into practice more effectively, cultivating research capabilities foundational to innovation.

The rapid development of positive psychology in modern high-pressure, high-intensity work environments has drawn extensive scholarly attention to the concept of positive variables. Thriving at work is a key concept in positive organizational behavior, describing the continuous psychological states of vigor and learning that individuals experience at work, in which they feel energized and acquire new skills, thereby enhancing their development and performance. Vigor, the affective dimension of thriving at work, refers to an individual’s positive feeling of having available energy. Learning, the cognitive dimension, pertains to an individual’s sense of acquiring and effectively applying knowledge and skills [30, 31]. Numerous studies have shown the positive effects of thriving at work on nurses. Liu et al. [25] found that nurse leaders with a platform leadership style can facilitate the demonstration of innovative behaviors by enhancing subordinate nurses’ thriving at work, based on a survey of 428 nurses. Thriving at work enhances nurses’ job performance and reduces work withdrawal behaviors [32, 33]. With the increasing Thriving at Work of clinical nurses, they will, on the one hand, have great enthusiasm for their work and the goals of the department and the hospital, and become more devoted to them; on the other hand, they will feel their actual growth and thus have a stronger sense of self-efficacy for the development of innovative work. Although the correlation between thriving at work and innovative behaviors has been confirmed, few studies have examined this relationship among ICU nurses. Within the high-stress context of the ICU, thriving at work is likely a significant factor influencing ICU nurses’ innovative behavior.

A growing number of scholars are focusing on spiritual climate, and the inquiry into how to cultivate a favorable one in the workplace has emerged as a prominent research topic across multiple disciplines. Spiritual climate refers to an organization that respects and encourages employees to express their inner thoughts, fostering a spiritual culture that directly affects employee work engagement [34, 35]. By increasing employee engagement at work, employees can be motivated to perform their jobs more effectively, thereby promoting innovation [36]. In a 2024 study, Zhang et al. [26] found a significant positive correlation between spiritual climate and the innovative behaviors of ICU nurses. Moreover, studies by Hashemian et al. [20] and Lu et al. [27] have confirmed that work environment factors influence nurses’ innovative behavior, and that an excellent organizational climate enhances nurses’ level of innovative behavior. These results provide a theoretical framework for understanding how the spiritual climate influences innovative behavior.

Although the correlations among nurses’ information literacy, spiritual climate, thriving at work, and innovative behavior have been confirmed, few studies have integrated these factors specifically among ICU nurses. In the complex and high-acuity ICU environment, the interplay of these variables may manifest in distinct patterns that influence innovative behaviors differently than in general ward settings. In addition, few studies have systematically ranked the importance of factors identified as influencing ICU nurses’ innovative behavior. The random forest model is a commonly used machine learning method for variable screening. By constructing multiple decision trees into an integrated learning framework, it can effectively handle complex, nonlinear relationships. It can accurately rank the importance of influencing factors and is highly efficient when dealing with large datasets and high-dimensional features [37, 38]. LASSO regression can exclude unimportant variables, greatly simplify the relationship between dependent and independent variables, prevent overfitting, and provide a reliable technical approach for the multifactorial analysis of ICU nurses’ innovative behaviors. Based on this, this study employs a Lasso regression model to identify key factors associated with innovative behaviors among ICU nurses. It applies a random forest algorithm to rank the relative importance of these factors, thereby providing evidence-based insights for the development of targeted, individualized interventions to promote innovation in ICU nursing practice.

## Objectives and methods

### Study design

We conducted a cross-sectional study on China ICU nurses. A STROBE checklist was used as the guideline in this study.

### Participants

This study involved 587 ICU nurses from twenty-three grade 2 A and above hospitals in Anhui Province, China, who were selected using a convenience sampling method. The inclusion criteria were as follows: (1) Obtained a professional qualification certificate for nurses; (2) Worked in an ICU for at least 6 months; (3) Given informed consent and voluntarily participated in this study. Exclusion criteria: Non-working nursing staff during the survey period. According to the sample size estimation requirements for multiple linear regression analysis studies, the sample size for the study was calculated using an estimation method that requires the sample size to be ten to twenty times the number of questionnaire items [39]. This study included a total of 23 independent variables. Taking into account a 20% loss in the visit rate, the required sample size was calculated to be between 276 and 552 cases. The actual survey was conducted with 587 ICU nurses, which met the sample size requirement.

### Data collection and survey instruments

The questionnaires used in this study included sociodemographic information, the Spiritual Climate Scale, the Information Literacy Scale, the Thriving at Work Scale, and the Innovative Behaviour Inventory.

#### Participant demographic information questionnaire

The questionnaire was drafted following discussions among research team members and primarily comprises 11 questions: gender, age, hospital rank, job nature, current position, education level, professional title, total years of nursing experience, whether or not you are an ICU nurses specialist, the degree of love for nursing work, and physical health.

#### Thriving at work scale

The Thriving at Work Scale was developed by Porath et al. (2012) [31] and translated and revised by Han et al. (2020) [40] to measure the level of Thriving at Work of employees, including two dimensions, the learning dimension (5 items) and the vitality dimension (5 items), with a total of 10 entries, each of which is scored on a 7-point Likert scale ranging from “strongly disagree” to “strongly agree” on a scale of 1 to 7, with entries 4 and 8 being reverse-scored entries. The total score ranges from 10 to 70, with higher scores indicating a higher level of Thriving at Work. The Cronbach’s alpha value for this scale is 0.771. The Cronbach’s alpha coefficient of this scale in this study was 0.902 for the overall scale, and the Cronbach’s alpha coefficients of the learning and vitality dimensions were 0.776 and 0.873, respectively.

#### Information literacy scale

The Information Literacy Scale, created by Shu (2009) [29] comprises four dimensions: information consciousness (4 items), information ability (6 items), information knowledge (6 items), and information morals (4 items), totaling 20 items. A 5-point Likert scale was employed, with “totally disagree” assigned 1 point and “totally agree” assigned 5 points. The total score ranged from 20 to 100, with higher scores indicating greater information literacy among nurses. The reliability (cronbach alpha) coefficient of the original form of the scale was found to be 0.911 [41]. The overall Cronbach’s alpha coefficient for the scale in this study was 0.967, while the coefficients for the dimensions of information consciousness, information ability, information knowledge, and information morals were 0.846, 0.952, 0.915, and 0.879, respectively.

#### Spiritual climate scale

The “Spiritual Climate Scale ” developed by Doram et al. (2017) [42] and adapted into China by Wu (2019) [43] was used to measure spiritual climate among healthcare workers. The scale consists of four items and one dimension, which are: I am encouraged to express spirituality in this clinical area; My spiritual views are respected in this clinical area; My spirituality has a comfortable home in this clinical area; and A diverse set of spiritual views are accepted in this clinical area. The Likert 5-point scale was used, with all items being positive questions, ranging from “strongly disagree” to “strongly agree,” scored from 1 to 5 points respectively. The average score of the four questions was subtracted by 1 and multiplied by 25 to obtain the final score, with a total score ranging from 0 to 100 points, where higher scores indicate better mental climate status among nurses. The Cronbach’s α value of the scale is 0.833. The overall Cronbach’s α coefficient of the scale in the present study was 0.964.

#### Innovative behavior inventory

Innovative Behavior Inventory (IBI) is a 20-item scale compiled by Martin Lukes and Ute Stephan (2017) [44]. The Chinese version scale was revised by Huang et al. (2021) [45], including five dimensions: idea generation and search(6 items), plan communication and implementation(5 items), involving others (3 items), overcoming obstacles(3 items) and clinical application (3 items), with a total of 20 entries. Each item was rated on a 5-point Likert scale from “not at all” to “strongly agree,” with a total score of 15 to 75, with higher scores indicating higher levels of innovative behavior. The Cronbach’s α coefficient for the Chinese version of the scale was 0.95 [46]. The overall Cronbach’s alpha coefficient for the scale in this study was 0.988. Finally, the reliability (cronbach’s alpha) coefficient for each dimension of the scale were determined as 0.970, 0.958, 0.955, 0.944, and 0.948, respectively.

### Random forest algorithm

The core idea of the Random Forest model is to calculate the model accuracy by building multiple decision trees, each based on modeling a different random subset of the training data, with the unextracted data serving as the test set. By integrating the predictions from multiple decision trees, the final prediction is typically selected through a voting process. The study ranks the importance of influencing factors based on mean decrease accuracy [37].

### Participants and data collection

Data was collected via the Chinese internet platform ‘Wenjuanxing’ (https://www.wjx.cn), which facilitates the creation of electronic questionnaires and the generation of matching QR codes. The cross-sectional survey method was used for data collection in this study. The survey was implemented by members of the team (including the first author and two uniformly trained investigators) through an electronic questionnaire platform. The researchers contacted the nursing management personnel of the hospital to provide a detailed explanation of the study’s purpose and significance, as well as obtain their consent. A head of the ICU nursing unit was designated as the survey leader in each of the twenty-three hospitals, with clear communication regarding the study’s objectives, voluntary participation principle, respondent inclusion/ exclusion criteria, anonymity principle, guidelines for answering questions and data collection specifications. The study protocol was communicated to the nursing staff of the undergraduate unit by the head nurse, focusing on the principles of anonymity, single-device IP-limited answers, and the breakpoint renewal function. The research team monitored the dynamics of questionnaire submission in real-time through the back-end management system and set 72 consecutive hours without new data as the survey termination point. The data quality control link utilizes a two-person blind checking mechanism to exclude four questionnaires that were not completed or exhibited irregular responses. A total of 591 electronic questionnaires were distributed, and 587 valid questionnaires were ultimately included, resulting in an effective response rate of 99.33%.

### Ethics approval and consent to participate

This study was conducted following the ethical principles required in the Declaration of Helsinki and approved by the ethical committee of the First Affiliated Hospital of the University of Science and Technology of China (ethical approval number: 2025-ky222). All participants were informed of the research objective, volunteered to participate in this survey, and had the right to refuse participation at any time. Informed consent was obtained from all participants. The study data are strictly confidential and are for research use only.

### Statistical analysis

The results were exported directly from Wenjuanxing and analysed using SPSS 22.0. There were no missing values for all available data, as required fields were set at the time of data collection. Measurement data conforming to a normal distribution were expressed as mean ± standard deviation. Independent sample t-tests and one-way ANOVAs were used for comparison between groups, and the Pearson method was chosen for correlation tests. Taking the total number of innovative behaviors as the dependent variable, the statistically significant indicators (*P* < 0.05) from the one-way analysis of variance and correlation test were used as the set of predictor variables. Firstly, the randomForest program package in R Studio 4.3.2 software was used to construct a random forest model comprising 500 decision trees to obtain variable importance scores. At the same time, the glmnet package was used to implement LASSO regression for feature dimensionality reduction. Finally, the common predictors identified by the two algorithms were incorporated into the multiple linear regression model, and the prediction equations were established using the stepwise regression method. Differences were deemed statistically significant at *p* < 0.05.

## Results

### Comparison of nursing innovation behaviour inventory among ICU nurses with different characteristics

Consequently, 587 valid questionnaires were obtained, resulting in an effective response rate of 99.33%. The age range of the participants was 20–56 years, with a mean age of 31.63 years (SD 5.352). There were 105 males and 482 females. This study categorized ICU nurses and compared their innovative behavior ratings based on demographic parameters, occupational characteristics, and physical health. Only statistically significant items are listed in this study. For more information, see Table 1.

**Table 1:** Demographic characteristics and innovation behaviour of ICU nurses

Demographic characteristics	*N*(%)	Score (M ± SD)	F/t	*P*
Age (years old)			7.684	<0.001
≤ 25	56	67.21 ± 17.989		
26∼30	229	76.09 ± 15.093		
31∼35	193	78.02 ± 14.102		
36∼40	73	77.92 ± 10.686		
≥ 41	36	81.42 ± 10.149		
Education level			24.203	<0.001
Junior college or below	101	67.60 ± 17.180		
Bachelor’s	476	78.34 ± 13.341		
Master’s or above	10	74.50 ± 16.188		
Professional title			12.219	<0.001
Nurse	68	68.15 ± 17.638		
Senior nurse	245	75.87 ± 14.610		
Nurse-in-charge	249	78.41 ± 13.238		
Deputy chief nurse or above	25	84.76 ± 9.628		
Current position				
Clinical nurse	532	75.47 ± 14.862	-8.087	<0.001
Head Nurse	55	85.73 ± 8.102		
Total years of nursing experience (years)			8.221	<0.001
<6	145	72.88 ± 16.634		
6∼10	211	75.11 ± 15.213		
11∼15	163	79.28 ± 12.674		
≥ 16	68	81.28 ± 9.840		
Physical health			6.554	0.002
Good	406	77.55 ± 14.813		
Fair	169	74.59 ± 13.353		
Worse	12	64.58 ± 20.170		
The degree of love for nursing work			82.429	<0.001
Like	219	83.45 ± 14.500		
More like	249	76.86 ± 9.789		
Not sure	79	66.78 ± 8.804		
Don’t like	40	54.43 ± 18.174		

### Descriptive statistics of thriving at work, information literacy, spiritual climate and innovation behaviour

In this study, the total scores for Thriving at Work Scale were (54.33 ± 10.15), for Information Literacy Scale were (76.39 ± 12.94), for Spiritual Climate Scale were (71.53 ± 19.82), and for Innovation Behaviour were (76.43 ± 14.67) (Table 2).

**Table 2:** Scores of thriving at work, information literacy, spiritual climate, and innovation behaviour (*n* = 587)

Variables	Items	Scoring range	Score X ± S	Average score X ± S
Thriving at work	10	12–70	54.33 ± 10.15	5.43 ± 1.01
Information literacy	20	22–100	76.39 ± 12.94	3.82 ± 0.65
Spiritual climate	4	0-100	71.53 ± 19.82	17.88 ± 4.96
Innovation behaviour	20	20–100	76.43 ± 14.67	3.82 ± 0.73
Idea generation and search	6	1–5	23.05 ± 4.54	3.84 ± 0.76
Plan communication and implementation	5	1–5	19.07 ± 3.78	3.81 ± 0.76
Overcoming obstacles	3	1–5	11.55 ± 2.29	3.85 ± 0.76
Clinical application	3	1–5	11.45 ± 2.27	3.82 ± 0.76
Involving others	3	1–5	11.31 ± 2.38	3.77 ± 0.79

### Correlation analysis of thriving at work, information literacy, spiritual climate and innovation behaviour

The correlation analysis results indicate significant positive correlations among Thriving at Work, Information Literacy, Spiritual Climate, and Innovation Behaviour. Thriving at work is positively correlated with Innovation Behaviour (*r* = 0.618, *p* < 0.001); thriving at work is positively correlated with information literacy (*r* = 0.723, *p* < 0.001); thriving at work is positively correlated with Spiritual Climate (*r* = 0.780, *p* < 0.001); information literacy is positively correlated with Innovation Behaviour (*r* = 0.627, *p* < 0.001); information literacy is positively correlated with Spiritual Climate (*r* = 0.706, *p* < 0.001); Spiritual Climate is positively correlated with Innovation Behaviour (*r* = 0.652, *p* < 0.001) (Table 3).

**Table 3:** Correlation coefficients among thriving at work, information literacy, spiritual climate, and innovation behaviour (*n* = 587)

Variables	1	2	3	4
Thriving at work	1.000			
Information literacy	0.723^**^	1.000		
Spiritual climate	0.780^**^	0.706^**^	1.000	
Innovation behaviour	0.618^**^	0.627^**^	0.652^**^	1.000

**p < 0.001, indicating significance in correlation

### Screening of factors influencing innovative behavior of ICU nurses

#### Ranking of variable importance

**Fig. 1:**
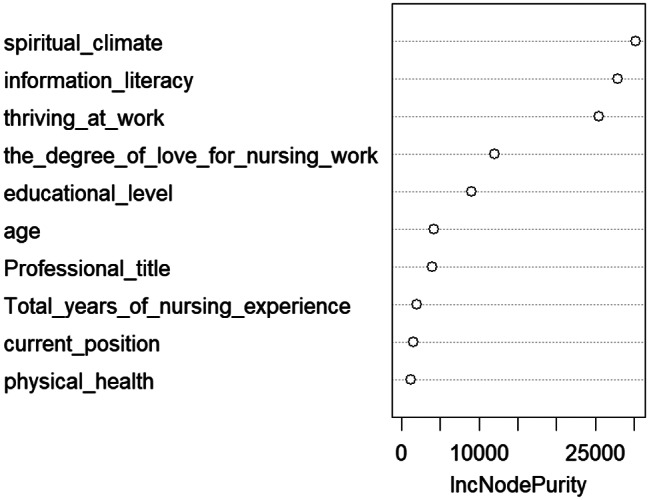
Importance ranking of factors influencing innovative behaviors in ICU nurses

In this study, the dependent variable was the ICU nurses’ innovative behaviour scores, and ten statistically significant variables identified in univariate and correlation analyses were selected for inclusion in the random forest model. The results were output using the Random Forest package in RStudio. Increase in Node Purity (IncNodePurity) measures the extent to which a feature (variable) contributes to reducing node ‘impurity’ when splitting nodes in a decision tree. The larger the value of IncNodePurity, the more important the feature is in increasing node purity (i.e., the degree of aggregation of samples of the same category) when splitting the data. The larger the IncNodePurity value, the more critical the feature [47]. The assignment situations of each variable in the random forest model are detailed in Table 4. The results showed that the spiritual climate variable had the highest importance, while the physical health status variable had the lowest importance, as illustrated in Fig. 1.

**Table 4:** Variable encoding and assignment in the random forest model

Variable	Code	Variable Assignment
Age	X1	≤ 25 years = 1, 26–30 years = 2, 31–35 years = 3, 36–40 years = 4, ≥ 41 years = 5
Education level	X2	Junior college or below = 1, Bachelor’s = 2, Master’s or above = 3
Professional title	X3	Nurse = 1, Senior nurse = 2, Nurse-in-charge = 3, Deputy chief nurse or above = 4
Current position	X4	Clinical nurse = 1, Head nurse = 2
Total years of nursing experience (years)	X5	<6 years = 1, 6–10 years = 2, 11–15 years = 3, ≥16 years = 4
Physical health	X6	Good = 1, Fair = 2, Worse = 3
The degree of love for nursing work	X7	Like = 1, More like = 2, Not sure = 3, Don’t like = 4
Thriving at Work	X8	Original value input
Spiritual Climate	X9	Original value input
Information Literacy	X10	Original value input

#### Variable selection

**Fig. 2:**
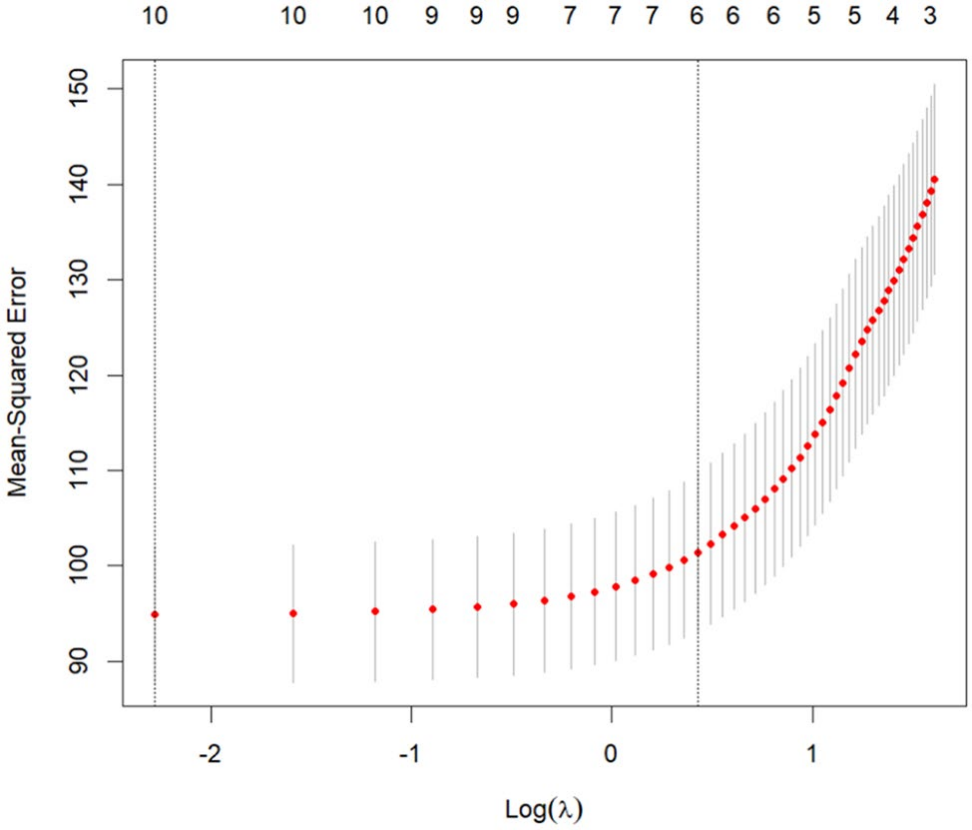
LASSO regression with cross-validation

Ranking results based on the importance of the variables, Lasso regression analysis was performed in R Studio using the glmnet function on 10 variables identified as statistically significant in the univariate analysis. As shown in Fig. 2, the vertical dashed line on the left represents lambda. min, while the line on the right represents lambda.1se. When the lambda (λ) value was 1.531, the model achieved the minimum error, corresponding to six influencing factors. Consequently, the top six variables—spiritual climate, information literacy, thriving at work, the degree of love for nursing work, educational level, and age—were included in the multivariate stepwise regression analysis.

#### Multivariate analysis of influencing factors of innovative behaviors of ICU nurses

Taking the total score of ICU nurses’ innovative behaviors as the dependent variable and combining the results of LASSO regression, the independent variables screened by the random forest model with the top 6 importance were included in the multivariate stepwise linear regression analysis. The results showed that spiritual climate, information literacy, thriving at work, the degree of love for nursing work, educational level, and age were the main influencing factors of ICU nurses’ innovative behaviors (all *p* < 0.05), which could explain 55.7% of the variance in innovative behaviors. See Table 5.

**Table 5:** Multiple Stepwise regression results of factors influencing innovative behavior of ICU nurses (*n* = 587)

Factor	B	SE of B	Beta	t	*P*-value
Constant	16.760	4.384	-	3.823	<0.001
Thriving at work	0.154	0.070	0.107	2.195	0.029
Spiritual climate	0.222	0.036	0.301	6.254	<0.001
Information literacy	0.295	0.049	0.260	6.047	<0.001
Age	1.337	0.415	0.093	3.218	0.001
Educational level	7.284	1.027	0.202	7.096	<0.001
The degree of love for nursing work	-2.173	0.598	-0.130	-3.637	<0.001

B Regression coefficient, SE of B Standard error of B, Beta Standardized beta coefficient, *R* = 0.750, R^2^ = 0.562, adjusted R^2^ = 0.557, F = 124.035, *P* < 0.001

## Discussion

Intensive care units possess distinct workforce profiles and specialized professional capacities, utilizing advanced technologies to manage critically ill patients. Nurses must be innovative, especially in specialized and complex areas, such as intensive care [48]. The total score of Innovation Behaviour among ICU nurses in this study was (76.43 ± 14.67), with an average score of all items at (3.82 ± 0.73), which is at a moderate to high level compared to the median score of 3.00 on the scale. These scores exceeded those reported by Wang et al. [49]. for psychiatric nurses and Hashemian et al. [50] for clinical nurses, suggesting ICU nurses generally possess substantive innovative capacity. Several factors may explain these findings. First, China’s national innovation-driven development strategy and enhanced healthcare innovation ecosystems have elevated nursing innovation levels, aligning with empirical evidence for online psychological capital interventions and transformational leadership theories in nursing interventions [51, 52]. Second, this study employed the IBI-20 scale, selected for its predictive validity and comprehensive measurement, which differs from the instruments used in prior research. Finally, ICU-specific characteristics (e.g., rapid technological iteration, high-risk clinical environments) necessitate continuous knowledge updating, which may foster greater innovation than in non-acute settings. The findings of study, Zhang, et al. [26] showed that the practical wisdom developed by ICU nurses in the process of coping with the complex conditions of acutely ill patients not only provides empirical evidence for the development of the nursing discipline but also promotes the innovation and optimization of the nursing education model through knowledge translation. Among the five dimensions of the ICU nurses’ Innovative Behavior Scale assessed in this study, the relatively high scores observed in the “Overcoming Obstacles” and “Idea Generation and Search” dimensions can be attributed to the higher educational attainment of the participating ICU nurses. Specifically, 82.8% of the nurses held a bachelor’s degree or higher. An increased level of education has been positively associated with enhanced critical thinking and clinical decision-making abilities, which may encourage nurses to engage in autonomous reasoning and adopt creative strategies when addressing clinical challenges [24]. This cognitive flexibility likely facilitates both the generation of innovative ideas and the capacity to overcome barriers in the pursuit of clinical objectives. Nursing managers should actively encourage ICU nurses to enhance their educational background. Conversely, the lower scores obtained in the “Involving others” dimension may be linked to the heavy workloads commonly experienced by ICU nurses, as well as a perceived lack of organizational support for implementing innovative nursing practices [53]. This discrepancy may be attributed to the influence of transformational leadership [54], due to a lack of effective team cooperation skills, they face difficulties in establishing their own innovative teams; consequently, they often undertake scientific research tasks independently resulting in a lower score for involving others dimension. Previous studies have indicated that organizing team collaboration and allocating tasks efficiently can save time while avoiding disruptions to daily nursing duties and facilitating successful completion of scientific research innovations [55]. Therefore, nursing managers should actively foster an innovative cultural atmosphere, carry out theoretical and practical innovative training teaching based on the ADDIE teaching model [56] and also form a professional nurse innovation service team to investigate and analyze nurses’ innovative ideas, seek available resources for them, assist in formulating implementation plans, and conduct utility evaluations to promote the realization of nurses’ innovative ideas. At the same time, an innovation reward system has been established, with sufficient attention and organizational support given to encourage nurses to take the initiative and continuously promote their learning, practice, and innovation.

The results of this study showed that the Spiritual Climate of the work environment was the most critical factor influencing the innovative behavior of ICU nurses (*β* = 0.222, *P* < 0.05). It was able to positively promote innovative behavior, which is consistent with the findings of He et al. [57]. The Conservation of resources Theory, proposed by Hobfoll [58], a positive work environment Spiritual Climate (e.g., teamwork, leadership empowerment, and organizational support) is a core conditioning resource that can lead to ICU nurses not needing to consume additional resources to cope with interpersonal conflict or managerial stress and can devote more energy to innovative behaviors. Previous studies support this notion: a favorable Spiritual Climate directly affects clinical nurses’ work commitment and job satisfaction, which in turn prompts them to be able to devote more time and energy to optimizing the functional framework of the position and the elements of the operational process, etc., and to innovate to enhance the level of innovation continuously [34, 59]. Xie et al. [60] suggested improving the Spiritual Climate of the working environment for nurses by enhancing teamwork. It is suggested that managers encourage ICU nurses to actively express their innovative ideas and spiritual needs, promote trust and effective communication among team members, proactively share innovative knowledge and experiences, facilitate the sharing of tacit knowledge within the team, and stimulate more innovative behaviors among nurses.

The findings of this study revealed that information literacy was the second most influential factor affecting the innovative behavior of ICU nurses (*β* = 0.295, *P* < 0.05). Information literacy can positively impact the innovative behavior of ICU nurses, which is consistent with studies by Zhong et al. [61] investigating nursing students and Wang et al. [24] examining specialized nurses. Information literacy, as a core competency for nurses’ lifelong learning and professional development, plays a fundamental role in innovative behaviors, enabling them to accurately retrieve and utilize information, evaluate its credibility and authority, and integrate practical information into their innovative ideas and approaches [61, 62]. Nurses who possess good information literacy skills can not only recognize the value of information but also locate relevant knowledge resources through professional information platforms and apply critical thinking to assess the truth, usefulness, and authenticity of information in various areas. In this mental process, nurses add verified information to their professional knowledge base, use reverse thinking to break down existing problems, and employ forward-looking thinking to explore new ways of doing things. They then realize the innovation of the nursing knowledge base by reassembling information, which leads to new technologies, methods, and ideas that are useful in real-life applications. This suggests that when hospital administrators consider the factors influencing nurses’ innovative behaviors, they can enhance nurses’ innovative behaviors by improving their information literacy. An ongoing education office can be established to systematically develop and implement information literacy training programs tailored to the needs of clinical nurses [63], integrating various forms of learning such as virtual training platforms [64], and scenario simulation [65] to enhance their level of information literacy.

This study reveals that Thriving at Work is the third major factor influencing the innovative behavior of ICU nurses (*β* = 0.154, *P* < 0.05), and it can positively promote innovative behavior. Nurses with a high level of Thriving at Work will deepen their understanding of clinical problems through continuous learning, come into contact with new ideas and perspectives, broaden their thinking horizons, and facilitate the identification of problems and the search for solutions, thus enhancing innovative behaviors. This finding is consistent with that of Liu et al. [25], who concluded that nurses with increasing Thriving at Work will experience stronger self-efficacy in carrying out innovative work due to their personal growth, which will encourage them to exhibit more innovative behaviors. The study carried out by Nguyen and McGuirk et al. [66] in Vietnam reported that when the level of Thriving at Work of nurses is high, individuals deepen their understanding of clinical problems through continuous learning, come into contact with more new concepts and perspectives, broaden their horizons of thinking, are better able to identify problems and seek solutions, and their innovative behavior is enhanced. The findings of Bai et al.‘s [67] study in China indicate that focusing on strengths promotes continuous learning and growth, fostering a sense of thriving at work. It is recommended that nursing administrators or leaders pay attention to differences in individual levels of strengths use, encourage strengths-based practices, and design interventions that foster hope, thereby promoting greater thriving in their professional roles [68]. In addition, nursing managers draw on magnetic hospital management strategies to build flexible scheduling systems, reasonable empowerment mechanisms, and hierarchical training systems in the clinical work environment, simultaneously guaranteeing the optimization of the practice environment and the maintenance of organizational fairness, as well as multi-dimensional interventions to improve the level of nurses’ work prosperity and ultimately stimulate their clinical innovation effectiveness.

One major contribution of our study was to find that these demographic characteristics did not uniformly impact innovative behavior, as we linked the selected demographic characteristics to innovative behavior using a random forest. For example, the results of this study show that the demographic characteristic factors influencing the innovative behavior of ICU nurses are, in order, the degree of love for nursing work, educational level, and age. Positive attitudes toward nursing work help promote innovative behavior. This study shows that the degree of love for nursing work is a key factor influencing the innovative behavior of ICU nurses, and the more positive the attitude toward nursing work, the higher the score of innovative behavior (*β* =-2.173, *P* < 0.05). According to Masaoud’s study [69], the degree of fondness for nursing work reflects the nurses’ work attitude to a certain extent and also determines the nurses’ efforts. The findings of this study also indicate that educational level is directly related to the level of innovative behavior of ICU nurses participating in this study. In line with this study, Xiang et al. [70] in China and Dayan et al. [48] in Turkey revealed that highly educated nurses may stem from their advantages in systematic knowledge reserves and theory-practice transformation capabilities. They possess stronger abilities in identifying clinical problems and generating innovative thinking, and are more likely to access support resources for innovative practices. Furthermore, the results of this study revealed that age is a significant factor influencing the innovative behavior of ICU nurses (*β* = 1.337, *P* < 0.05). Other studies, in line with the findings of this study, have noted that senior nurses possess rich clinical experience and exhibit active, innovative thinking. It is easier to form the output ability of scientific research results driven by the demand for title promotion. In contrast, junior nurses are often faced with insufficient organizational support, time and resource constraints, and environmental constraints due to seniority limitations, which makes it difficult to implement their innovative programs effectively and ultimately leads to a low rate of innovation transformation [71]. This study suggests that nursing managers should prioritize nurses’ career development needs and enhance work motivation by listening to their expressions of work attitudes and understanding their career motivations and goals. This study reveals that only 1.70% of clinical nurses hold a master’s degree, and the lack of continuing education may hinder the development of their innovative abilities. It is recommended that a tiered training system be constructed based on the differences in nurses’ seniority and competence levels, that innovative courses be designed to match their positions, and that an innovative mentorship system be developed for highly educated and senior nurses to provide sustainable support for the innovative practice of nursing teams.

## Limitations

This study has several limitations that need to be considered. First, the questionnaire utilized for this survey was self-reported by ICU nurses, potentially resulting in findings that are somewhat inflated compared to the actual levels. To mitigate this bias, subsequent researchers may employ evaluations conducted by others or utilize interviews. Second, this is a cross-sectional survey, which may limit the ability to infer causal relationships. Future longitudinal studies may investigate the long-term effects and causal relationships among these variables. Third, the relational paradigm in this research requires validation across diverse demographics and urban areas. Finally, this study employed a restricted set of fundamental personal and professional characteristics as independent variables, potentially overlooking significant elements such as methods of continuing education. Future studies should meticulously investigate the aspects related to continued education.

## Conclusion

This multicentre cross-sectional study, utilizing a random forest model combined with multivariate stepwise linear regression, identified and ranked key factors influencing innovative behaviours among ICU nurses in China. Our findings demonstrate that ICU nurses exhibit moderately high levels of innovative behaviour. Spiritual Climate, Information Literacy, and Thriving at Work emerged as significant positive predictors. The degree of love for nursing work, education level, and age were also identified as influential factors, ranked according to their relative importance by the random forest algorithm. These results provide crucial insights for nurse managers and healthcare leaders. Specifically, developing targeted interventions to enhance spiritual climate, foster information literacy, and promote thriving at work offers a strategic pathway to support and amplify innovative practices among ICU nurses within the healthcare context.

## Practical implications of the study

This research is the first to use a random forest model to analyse factors associated with ICU nurses’ innovative behaviours, offering a fresh perspective compared with linear or logistic regression studies. The findings of this study have significant practical implications in several aspects: Firstly, the innovative behaviour of Chinese ICU nurses is at a moderate to high level. Nursing managers should recognize that enhancing innovative behaviour has crucial implications for nursing quality and industry development. Organizations should formulate innovation-centric policies to motivate nursing leaders to support innovation and foster an environment that promotes new practices [72]. Secondly, nursing managers should promote the information literacy of ICU nurses to enable them to effectively acquire, evaluate, and utilize information, thereby establishing a strong foundation for nursing innovation. Nursing administrators can expand nurses’ innovative information acquisition and access by carrying out professional courses such as resource and interactive exchange platforms, scientific research lectures, and literature searches, establish a scientific information literacy cultivation system for nurses, and cultivate their critical and dialectical thinking, and promote their innovative behaviors in the process [73]. Thirdly, nursing managers pay attention to nurses’ work emotions, monitor their work status, and timely understand and defuse negative work emotions. This helps build a spiritually friendly, comfortable, and safe work culture atmosphere, stimulates nurses’ work enthusiasm and initiative, and ultimately improves work performance and innovation levels. Finally, leaders should prioritize shaping an empowering work environment to enhance the innovation efficiency of nursing work. For example, establishing a fair and equitable system and evaluation process, building a feedback environment, and providing mental health resources, as well as more opportunities for career development, are ways to enhance nurses’ Thriving at Work [74], which in turn promotes innovative behaviors.

## Data Availability

The data that support the findings of this study are available on request from the corresponding author. The data are not publicly available due to privacy or ethical restrictions.

## Declarations

### Impact

This study provides the first quantitative evidence on the relative importance of modifiable factors (spiritual climate, information literacy, and thriving at work) that drive innovative behaviors among intensive care nurses in China, using random forest modeling. Findings offer nurse administrators actionable priorities, including implementing tiered information literacy training, enhancing the Intensive Care Unit spiritual climate, facilitating interventions for thriving at work, and leveraging senior nurses’ expertise, to systematically develop innovation-supportive environments that address critical care challenges through empowered nursing practice.

## Abbreviations

ICU: Intensive Care Unit
IBI: Innovative Behavior Inventory
LASSO: Least Absolute Shrinkage and Selection Operator
ADDIE: Analysis, Design, Development, Implementation, Evaluation

## References

[1] Al Wali J, Muthuveloo R, Teoh AP, Al Wali W. Disentangling the relationship between employees’ dynamic capabilities, innovative work behavior and job performance in public hospitals. Int J Innov Sci. 2023;15:368–84.

[2] Fleiszer AR, Semenic SE, Ritchie JA, Richer M-C, Denis J-L. The sustainability of healthcare innovations: a concept analysis. J Adv Nurs. 2015;71:1484–98. 25708256 10.1111/jan.12633

[3] Cheng L, Wei W, Zhang J, Zhang Y, Liu N. Clinical nurses experiences of innovative behavior: a qualitative study. J Nurs Sci. 2022;37:59–62.

[4] Drennan VM, Ross F. Global nurse shortages—the facts, the impact and action for change. Br Med Bull. 2019;130:25–37. 31086957 10.1093/bmb/ldz014

[5] Bao L, Wang L, Zhang Y-Q. Development and analysis of reliability and validity of nurse innovative behavior scale. J Shanghai Jiaotong University(Medical Science). 2012;32.

[6] Yan T, Ning P, Xu Y, Li S. Research progress on innovative behavior in nurses. J Nurs Sci. 2022;37(18):102–5.

[7] Asurakkody TA, Shin SY. Innovative behavior in nursing context: a concept analysis. Asian Nurs Res. 2018;12:237–44. 10.1016/j.anr.2018.11.003 30471386

[8] Abdelwahab Ibrahim El-Sayed A, Shaheen RS, Farghaly Abdelaliem SM. Collaborative leadership and productive work performance: the mediating role of nurses’ innovative behavior. Int Nurs Rev. 2024;71:868–78. 38217403 10.1111/inr.12934

[9] American Nurses Association. ANA Enterprise 2023–2025 Strategic Plan.2023. https://www.nursingworld.org/globalassets/ana-enterprise/about-us/ana-strategic-plan-2024.pdf

[10] National Health Commission. National Nursing Development Plan (2021–2025). Chinese Nursing Management. 2022;22(6):801–804.

[11] Labeau S, Chiche J-D, Blot S. Post-registration ICU nurses education: plea for a European curriculum. Int J Nurs Stud. 2012;49(2):127–8. 21868012 10.1016/j.ijnurstu.2011.07.014

[12] Sauro KM, Soo A, Quan H, et al. Adverse events among hospitalized critically ill patients: A retrospective cohort study [J]. Med Care. 2020;58(1):38. 31688552 10.1097/MLR.0000000000001238

[13] Shin JW, Choi J, Tate J. Interventions using digital technology to promote family engagement in the adult intensive care unit: an integrative review. Heart Lung. 2023;58:166–78. 36525742 10.1016/j.hrtlng.2022.12.004 PMC9750805

[14] Khurrum M, Asmar S, Joseph B. Telemedicine in the ICU: innovation in the critical care process. J Intensive Care Med. 2021;36:1377–84. 33111599 10.1177/0885066620968518

[15] Sabzevari S, Mirzaei T, Bagherian B, Iranpour M. Critical care nurses’ attitudes about influences of technology on nursing care. Br J Med Med Res. 2015;9(8).

[16] Talebi A, Ravari A, Mirzaei T, et al. Effect of nurse’s verbal communication on the level of consciousness, pain, and agitation in anesthetized patients admitted to the intensive care unit: a double-blind clinical trial. BMC Anesthesiol. 2025;25(1):240. 10.1186/s12871-025-03071-5. 40348962 10.1186/s12871-025-03071-5 PMC12066066

[17] Zhou T, Li C, Wang Z, et al. Evidence-Based practice in maintenance of central venous catheters among intensive care unit nurses: A Cross-Sectional Multi-Center study. J Clin Nurs. 2025;34(10):4351–65. 10.1111/jocn.17692. 40125637 10.1111/jocn.17692

[18] Gao H, Yang Y, Xu L, Zhang Y. Correlation research between occupational benefit and innovative behavior of ICU nurses [J]. Chin Nurs Res. 2016;30(14):1781–2.

[19] Duan X, Shen T, Xu W, Wu A, Xie H. Examining the mediating role of role identity and knowledge sharing in the association between leadership support and nurses’ innovative behaviour: a multicentre cross-sectional study. J Adv Nurs. 2025;1–13. 10.1111/jan.17038 40349116

[20] Hashemian M, Hashemian Moghadam A, Hosseini M, Azizpour I, Mirzaei A. Examining the relationship between workplace fun and innovative behavior among nurses: the mediating effect of innovation support and affective commitment. J Nurs Adm Manag. 2024;2024:9629172. 10.1155/2024/9629172 PMC11919014 40224838

[21] Li H, Qiao Y, Wan T, Shao CH, Wen F, Liu X. Profiles of innovative behavior and associated predictors among clinical nurses: a multicenter study using latent profile analysis. BMC Nurs. 2025;24:77. 39844113 10.1186/s12912-025-02716-7 PMC11756079

[22] Huang Y, Liu Y, Wen J. Status quo and influencing factors of innovative behavior of nurses in general hospitals. Chin Nurs Res. 2024;38(22):3997–4003.

[23] Middleton L, Hall H. Workplace information literacy: a Bridge to the development of innovative work behaviour. J Doc. 2021;77:1343–63.

[24] Wang Z, Wen N, Ren Y, Liang W, Ding Y, Ji M, et al. Mediating role of evidence-based nursing competence between specialist nurses’ information literacy and innovative behaviour: a multicentre cross‐sectional study. J Adv Nurs. 2025;81:2465–76. 39206870 10.1111/jan.16433

[25] Liu Y, Zhou H, Cao Q. The impact of platform leadership on innovative behavior of nurses under the background of high-quality development of public hospitals. Chin J Health Policy. 2024;17(3):72–8.

[26] Zhang Z, Qin H, Chen Y. The chain mediating effect of proactive personality and leisure crafting in ICU nurses on spiritual climate and innovative behavior. J Hunan Normal University(Medical Science). 2024;21(5):158–64.

[27] Lu Y, Zhai S, Liu Q, Liu J, Chen C. The impact of head nurse empowerment on clinical nurses’ innovative behavior: the mediating role of organizational climate and professional autonomy. BMC Nurs. 2025;24:574. 40399842 10.1186/s12912-025-03214-6 PMC12096589

[28] Ming H, Lin Z, Luo L, Huang S. The international comparison on the connotation and structure of information literacy. J Beijing Normal University(Social Sciences). 2019;02:59–65.

[29] Shu Y. “Investigation on the current situation of information literacy of postgraduates” Master’s degree. Suzhou University. 2009.

[30] Spreitzer G, Sutcliffe K, Dutton J, Sonenshein S, Grant AM. A socially embedded model of thriving at work. Organ Sci. 2005;16:537–49.

[31] Porath C, Spreitzer G, Gibson C, Garnett FG. Thriving at work: toward its measurement, construct validation, and theoretical refinement. J Organ Behav. 2012;33:250–75.

[32] Shen Z-M, Wang Y-Y, Cai Y-M, Li A-Q, Zhang Y-X, Chen H-J, et al. Thriving at work as a mediator of the relationship between psychological resilience and the work performance of clinical nurses. BMC Nurs. 2024;23:194. 38520023 10.1186/s12912-024-01705-6 PMC10958833

[33] Xu H, Wu C, Chen C, Yan B, Zha N, Zhang K, et al. The effect of illegitimate tasks on work withdrawal behavior of intensive care unit nurses: the chain mediating effect of perceived organizational support and thriving at work. Intensive Crit Care Nurs. 2025;90:104136. 40578249 10.1016/j.iccn.2025.104136

[34] Ashmos DP, Duchon D. Spirituality at work: A conceptualization and measure. J Manage Inq. 2000;9:134–45.

[35] Cruz JP, Alquwez N, Balay-odao E. Work engagement of nurses and the influence of spiritual climate of hospitals: A cross-sectional study. J Nurs Adm Manag. 2022;30:279–87. 10.1111/jonm.13492 34619805

[36] Uppathampracha R, Liu G. Leading for innovation: Self-efficacy and work engagement as sequential mediation relating ethical leadership and innovative work behavior. Behav Sci. 2022;12:266. 36004837 10.3390/bs12080266 PMC9405150

[37] Xu H, Li H, Fan Y, Wang Y, Li Z, Zhou L, et al. Analysis of factors influencing chemotherapy-induced peripheral neuropathy in breast cancer patients using a random forest model. Breast. 2025;81:104457. 40245641 10.1016/j.breast.2025.104457 PMC12144932

[38] SuWen L, YuYang H, Fengzhen W. Random forest analysis of ICU nurses’ knowledge, attitudes and practices in oral care for ventilator-associated pneumonia prevention. Nurs Crit Care. 2025;30:e13289. 39996334 10.1111/nicc.13289

[39] Norman G, Monteiro S, Salama S. Sample size calculations: should the emperor’s clothes be off the Peg or made to measure? BMJ. 2012;345. 10.1136/bmj.e5278 22918496

[40] Han Y, Liu G. Influence of authentic leadership and leader-member fitness on thriving at work: from social embedded perspective. J Bus Econ. 2020;03:28–40.

[41] Jiang R, Li X, Chen M, Lai C, Yang Y. The influence of nurses’information literacy and evidence-based nursing abilities on innovative behavior. J Nurs Sci. 2023;38(17):77–80.

[42] Doram K, Chadwick W, Bokovoy J, Profit J, Sexton JD, Sexton JB. Got spirit? The spiritual climate scale, psychometric properties, benchmarking data and future directions. BMC Health Serv Res. 2017;17:132. 28189142 10.1186/s12913-017-2050-5 PMC5303307

[43] Wu X, Zhang Y, Wu J, Wan X, Hu Y, Liu Y, et al. Study on reliability and validity of the Chinese version of the spiritual climate scale. Chin Nurs Res. 2019;33(14):2396–9.

[44] Lukes M, Stephan U. Measuring employee innovation: A review of existing scales and the development of the innovative behavior and innovation support inventories across cultures. IJEBR. 2017;23:136–58.

[45] Huang L, Chen J, Wang J, Zhang Y. Reliability and validity testing of Chinese version innovative behavior scale (lBl) in Chinese nurses. J Nurs Sci. 2021;36(07):81–4.

[46] Cheng L, Wei W, Zhang J, Yao Y, Zhang Y, Zhu W. The association of leader–member exchange and team–member exchange with nurses’ innovative behaviours: A cross-sectional study. J Adv Nurs. 2024;80:2813–21. 38482900 10.1111/jan.16121

[47] Zou L-X, Wang X, Hou Z-L, Sun L, Lu J-T. Machine learning algorithms for diabetic kidney disease risk predictive model of Chinese patients with type 2 diabetes mellitus. Ren Fail. 2025;47:2486558. 40195601 10.1080/0886022X.2025.2486558 PMC11983574

[48] Dayan A, Ince S. Individual innovative features of the intensive care nurses. Dimens Crit Care Nurs. 2025;44:137–44. 40163336 10.1097/DCC.0000000000000690

[49] Wang G, Shen Y, Ming L, Cui Y, Guan C. Research on current situation of innovative behavior among psychiatric nurses and its correlation with career development. Occupation Health. 2025;41(5):636–40.

[50] Hashemian Moghadam A, Nemati-Vakilabad R, Imashi R, Yaghoobi Saghezchi R, Mirzaei A. Psychometric properties of the Persian version of the innovative behavior inventory-20 items (IBI-20) in clinical nurses: a cross-sectional study. BMC Nurs. 2024;23:944. 39709430 10.1186/s12912-024-02634-0 PMC11663333

[51] Agaoglu FO, Bas M, Tarsuslu S, Ekinci LO. Serial mediating role of transformational leadership and perception of artificial intelligence use in the effect of employee happiness on innovative work behaviour in nurses. BMC Nurs. 2025;24:137. 39915749 10.1186/s12912-025-02776-9 PMC11800594

[52] Yan D, Chen L, Li M, Zhang Y, Zhang Y. Reducing anxiety and enhancing innovation in nurses: a psychological capital intervention study in China. BMC Nurs. 2025;24:204. 39987103 10.1186/s12912-025-02838-y PMC11847354

[53] Yang L, Fang J, Ye H, Li Y, Sun Q. Effects of competence in Evidence-Based nursing practice and perceived organizational support on nurses’innovative behavior. Military Nurs. 2021;38(11):54–7.

[54] Agaoglu FO, Bas M, Tarsuslu S, Ekinci LO. Serial mediating role of transformational leadership and perception of artificial intelligence use in the effect of employee happiness on innovative work behaviour in nurses. BMC Nurs. 2025;24(1):137. 39915749 10.1186/s12912-025-02776-9 PMC11800594

[55] Yan, Li M, Zhang Y, Zhang Y. A qualitative study of facilitators and barriers to nurses’ innovation at work. J Nurs Adm Manag. 2022;30:3449–56. 10.1111/jonm.13811 36121750

[56] Zhang Z, He X, Jiang Y, Wu L, Zhang L, Yan J. Using the ADDLE model of instructional design to teach nursing innovation and effect evaluation. J Nurs Sci. 2019;34(10):73–5.

[57] He Q, Wang J, Guo Y, Li J. The influence of nursing work environment and vocational delay of gratification on nurse innovative behavior in the tertiary grade A hospitals. J Nurs Adm. 2023;23(9):711–6.

[58] Hobfoll SE. Conservation of resources. A new attempt at conceptualizing stress. Am Psychol. 1989;44:513–24. 2648906 10.1037//0003-066x.44.3.513

[59] Lee E-H, Yu H-J. Effects of perceived spiritual management, work engagement, and organizational commitment on job satisfaction among clinical nurses: the mediating role of perceived spiritual management. BMC Nurs. 2023;22:462. 38057854 10.1186/s12912-023-01625-x PMC10699019

[60] Xie B, Wu X, Bi Y, He Y, Zhang Y. Current situation and influencing factors of nurses’spritual climate in tertiary comprehensive traditional Chinese medicine hospitals. Chin Evidence-Based Nurs. 2025;11(2):303–8.

[61] Zhong Z, Hu D, Zheng F, Ding S, Luo A. Relationship between information-seeking behavior and innovative behavior in Chinese nursing students. Nurse Educ Today. 2018;63:1–5. 29407253 10.1016/j.nedt.2018.01.004

[62] Yang L, Ye H, Sun Q, Fang J. Status quo of innovative behavior of clinical nurses and its influence factors: A 1,386-case study. J Nurs. 2022;29(2):57–61.

[63] Wu C, Zhang Y, Wu J, Zhang L, Du J, Li L, et al. Construction and application on the training course of information literacy for clinical nurses. BMC Med Educ. 2023;23:614. 37644432 10.1186/s12909-023-04505-9 PMC10466842

[64] Falahati-Marvast F, Sabzi A, Lotfalizadeh M, Farokhzadian J. Effectiveness of virtual training on nursing students’ intentions to engage in evidence-based practice: a case study in Iran. BMC Health Serv Res. 2025;25:650. 40329371 10.1186/s12913-025-12818-2 PMC12057094

[65] Chen S-H, Lin B-B, Wang X-Y, Xu G-R, Song J-H. Construction and implementation of a comprehensive midwifery skills practice course based on scenario simulation teaching: an action research. Nurse Educ Today. 2025;152:106754. 40288241 10.1016/j.nedt.2025.106754

[66] Nguyen NP, McGuirk H. Evaluating the effect of multifactors on employee’s innovative behavior in smes: mediating effects of thriving at work and organizational commitment. Int J Contemp Hospitality Manage. 2022;34:4458–79.

[67] Bai B, Qiao J, Bai C. Harnessing strengths from trauma: examining the impact of strength use on nurses’ job satisfaction, positive mental health, and thriving at work through post-traumatic growth. BMC Nurs. 2025;24:438. 40251504 10.1186/s12912-025-02936-x PMC12007320

[68] Wu J, Shen Z, Ouyang Z, Xiang Y, Ding R, Liao Y, et al. Strengths use and thriving at work among nurses: a latent profile and mediation analysis. BMC Nurs. 2025;24:69. 39833813 10.1186/s12912-025-02715-8 PMC11749353

[69] Cummings G, Olson K, Raymond-Seniuk C, Lo E, Masaoud E, Bakker D, et al. Factors influencing jobsatisfaction of oncology nurses over time. Can Oncol Nurs J. 2013;23(3):162–71. 24028035 10.5737/1181912x233162171

[70] Xiang D, Xu Y, Chen L, Zhang J, Yan L, Zhang J, et al. Nurses’innovative behavior and its influencing factors of 5 tertiary grade A hospitals in xi’an. Chin Nurs Manage. 2024;24(12):1804–8.

[71] Liu W, Chen H, Liu B, Wang Y, Huang C, Mo F, et al. Mediating role of innovation self-efficacy in the relationship between sense of organizational fairness and innovation behavior in nurses. Chin Occup Med. 2023;50(4):424–9.

[72] Singh SK, Gupta S, Busso D, Kamboj S. Top management knowledge value, knowledge sharing practices, open innovation and organizational performance. J Bus Res. 2021;128:788–98.

[73] Bao H, Wang Z, Zhao H, Zhu Y, Pang X, Hui C. Analysis on mediating effect of nurses’ information literacy between innovative behavior and organizational innovation climate in five tertiary grade-A hospitals in Tianjin. Occup Health. 2023;39(06):798–802.

[74] Jiang Y, Shen Z, Li P, Long W, Cai Y. Chain mediating effect of job crafting and thriving at work on nursing organizational support and innovati’ve behayior. Occup Health. 2025;1–6.

